# Development of a novel model of hypertriglyceridemic acute pancreatitis in mice

**DOI:** 10.1038/srep40799

**Published:** 2017-01-12

**Authors:** Yiyuan Pan, Yong Li, Lin Gao, Zhihui Tong, Bo Ye, Shufeng Liu, Baiqiang Li, Yizhe Chen, Qi Yang, Lei Meng, Yuhui Wang, George Liu, Guotao Lu, Weiqin Li, Jieshou Li

**Affiliations:** 1 Surgical Intensive Care Unit (SICU), Department of General Surgery, Jinling Hospital, Medical School of Nanjing University, No. 305 Zhongshan East Road, Nanjing 210002, Jiangsu Province, China; 2 Center for Immunology and Infectious Diseases, Biosciences Division, SRI International, Harrisonburg, VA 22802, USA; 3 Institute of Cardiovascular Science, Key Laboratory of Molecular Cardiovascular Science Ministry of Education, Institute of Cardiovascular Science, Peking University, Beijing, 100191, China; 4 Department of Gastroenterology, The Second Clinical Medical College, Yangzhou University, Yangzhou, China

## Abstract

The morbidity rate of hypertriglyceridemic acute pancreatitis (HTG-AP) increased rapidly over the last decade. However an appropriate animal model was lacking to recapitulate this complicated human disease. We established a novel mice model of HTG-AP by poloxamer 407 (P-407) combined with caerulein (Cae). In our study, serum triglyceride levels of P-407 induced mice were elevated in a dose-dependent manner, and the pancreatic and pulmonary injuries were much severer in HTG mice than normal mice when injected with conventional dose Cae (50 ug/kg), what’s more, the severity of AP was positively correlative with duration and extent of HTG. In addition, we found that a low dose Cae (5 ug/kg) could induce pancreatic injury in HTG mice while there was no obvious pathological injury in normal mice. Finally, we observed that HTG leaded to the increased infiltrations of macrophages and neutrophils in mice pancreatic tissues. In conclusion, we have developed a novel animal model of HTG-AP that can mimic physiological, histological, clinical features of human HTG-AP and it could promote the development of therapeutic strategies and advance the mechanism research on HTG-AP.

Acute pancreatitis (AP) is a common and devastating inflammatory condition of the pancreas that is considered to have the characteristics of acute onset, rapid progression and high mortality, and its annual incidence rate is about 700 per million^1^. What’s more, most AP could involve peripancreatic tissues and other distant organs, and then develop into serious secondary local and systemic complications, such as infected pancreatic necrosis (IPN), acute respiratory distress syndrome (ARDS), acute kidney injury (AKI) and sepsis. The main causes of AP include biliary tract disease, alcoholism, mechanical injury, hypertriglyceridemia (HTG), drug and infection^2^. Clinical researches in Europe have showed that biliary pancreatitis and alcoholic pancreatitis account for 37.1% and 41% of total incidence respectively^3^. With further studies for the etiology of AP, it was found that HTG has been the third major cause of AP following gallstone and alcohol over the last decade, and accounts for about 4–10% of incidence of total AP^4^,^5^. Especially in China, the morbidity rate could reach up to 15–20%^6^.

A study has showed that the onset risk of AP was about 5% when serum triglycerides (TG) level >1000 mg/dl, and increased dramatically up to 10–20% when the serum TG level >2000 mg/dl^7^. The current international consensus strongly suggests these AP patients with serum triglyceride level >1000 mg/dl to have hypertriglyceridemic acute pancreatitis (HTG-AP)^8^.

Compared with acute gallstone pancreatitis, HTG-AP has the characteristics of more complications and higher recurrence rate. The current literature on HTG-AP mainly focus on the analysis of clinical characteristics and there is less mechanism research that may be due to the lack of appropriate animal model for HTG-AP. The Lipoprotein Lipase (LPL) activity of mice and rats is so high that simply feeding high-fat diet can’t establish ideal animal model of HTG (TG level >1000 mg/dl). what’s more, currently reported genetically modified mice which used in the study of HTG-AP, such as LPL deficient mice^9^,^10^,^11^, human-apolipoprotein CIII transgenic (ApoCIII-tg) mice^12^,^13^ is difficult to get. Therefore, there is an urgent need to develop new HTG-AP animal models to promote the study of pathogenesis and specific prevention measures of HTG-AP.

Poloxamer 407 (P-407) is a hydrophilic triblock copolymer comprised of polyoxyethylene and polyoxypropylene units and has been reported to induce HTG with little side effects^14^. P-407 can increase the serum triglyceride concentrations by directly inhibiting the activity of both LPL and hepatic lipase, which were combined with the capillary wall^15^,^16^. Physiological toxicity of P-407 is so low that both short-term and long-term use can induce high serum triglyceride levels in mice^14^. Saja *et al*.^17^ found that serum triglyceride level of mice could rise up to 4000 mg/dl after treated with P-407 for 28 days and long-term HTG can cause lipid deposition in heart, liver and kidney with infiltration of macrophages and other pathological changes. Therefore we put forward that using P-407 to establish HTG model, then inducing AP by intraperitoneal injection caerulein (Cae) to build a HTG-AP mice model which provides feasibility for the mechanism study of HTG-AP.

## Results

### P-407 induced severe HTG in mice

**Figure 1:**
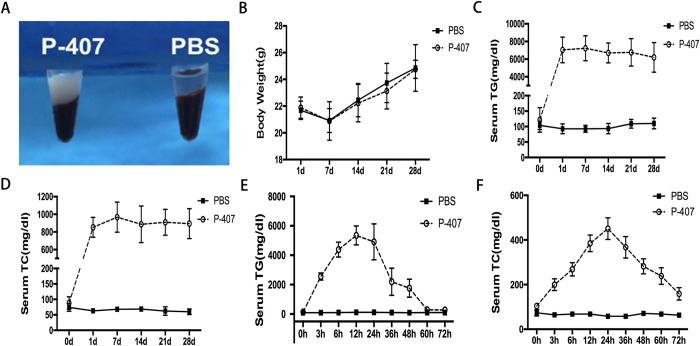
P-407 induced severe HTG in mice. P-407 group mice were administrated with long term 28 days P-407(0.5 g/kg) injections. n = 6–8 each group. (**A**) The serum of mice became obviously lactescent (milky coloration) after P-407 injection. (**B**) The body weight of mice in P-407 group and the PBS group. (**C**,**D**) The serum triglyceride and cholesterol levels of mice. (**E**,**F**) The change of serum triglyceride and cholesterol levels of mice after one single intraperitoneal injection of P-407 and the PBS group.

Consistent with the previous outcomes of Professor Saja^17^, we found that P-407 could elevate the serum ApoCIII levels which affected the metabolism of triglyceride and induced hypertriglyceridemia (Fig. S1A). The result of fast protein liquid chromatography (FPLC) further validated this phenomenon and indicated that hyperlipidemia in mice induced by P-407 was mainly composed of very low-density lipoprotein (Fig. S1C,D). After one single intraperitoneal injection of high dose (0.5 g/kg) P-407, serum triglyceride and cholesterol levels of mice increased rapidly and the peak values appeared around 12 h and 24 h after the injection respectively, then declined slowly and finally returned to normal values after 72 hours (Fig. 1E,F), While after the injections of low dose (0.1 g/kg, 0.25 g/kg) P-407, the peak values of HTG levels declined and metabolic elimination time had been moved up significantly (Fig. S2A,B). In addition, we observed mice HTG models by long-term P-407 induction in different doses (0.1 g/kg, 0.25 g/kg, 0.5 g/kg) and found that there was a positive correlation between the severity of hypertriglyceridemia and P-407 doses (Fig. 1C,D and Fig. S2C,D).

28 days after continuous intraperitoneal injection of 0.5 g/kg P-407, the serum of P-407 induced mice became obviously lactescent (milky coloration, Fig. 1A) and the serum triglyceride levels in more than 80% mice were higher than 6000 mg/dl (Fig. 1C), which were more than 50-folds higher than PBS control group. To investigate the safety of P-407, we examined the liver and kidney functions of the P-407 group, and continuously recorded the body weight of mice of the P-407 group and PBS group. P-407 treatment exerted no effects on body weight (Fig. 1B), as well as serum alanine transaminase level, serum creatinine level and other liver or kidney function indexes (Fig. S3).

### HTG aggravated pancreatic injury of AP

Firstly, in order to assess the effects of different extents of HTG exerted on AP, we adopted the above mentioned three doses P-407 to establish mice high triglyceride levels in three gradients. After induction of AP with standard dose Cae (50 ug/kg). we observed that AP severity was positively associated with serum triglyceride levels rather than the serum amylase levels (Fig. S5) and this was consistent with the clinical characteristics of HTG - AP^18^,^19^. In view of the most remarkable injury of mice AP induced by high dose P-407, hence, we adopted the dose (0.5 g/kg) as the follow-up experiment research dose.

**Figure 2:**
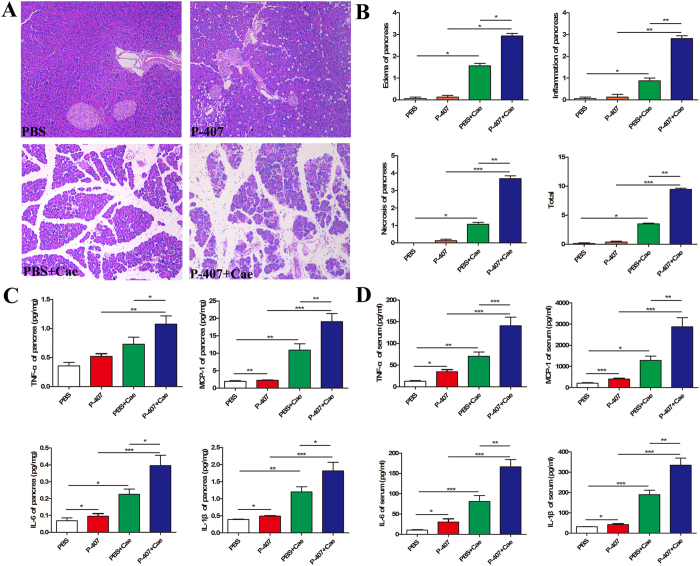
HTG aggravated pancreatic injury in mice. HTG was induced by long term 28 days P-407 (0.5 g/kg) injection, Mice was treated with standard dose Cae (50 ug/kg) to induce AP model. n = 6–8 each group. (**A**) Representative pathological changes in pancreas. HE stained sections of pancreas in magnification 100X. (**B**) Histological scores of pancreatic tissue. (**C**)Levels of TNF-a, IL-6, MCP-1, IL-1β in pancreatic tissues. (**D**)Serum levels of TNF-a, IL-6, MCP-1, IL-1β. n = 6–8 each group. *P < 0.05, **P < 0.01, *** < 0.001.

Next, to gain insights into the influence of different durations of HTG on AP, we divided HTG mice into three groups: transient 24 h HTG group induced by single intraperitoneal injection of P-407; short-term HTG group induced by the 7 days injection of P-407 and long-term HTG group induced by the 28 days injection of P-407. After the induction of standard dose Cae (50 ug/kg), compared with PBS+Cae group, all three groups of HTG can deteriorate the pancreatic injury degree. Transient HTG mice were mainly characterized as edema, inflammatory cells infiltration without obvious necrosis, while the necrosis of short-term and long-term HTG mice were significantly severer and the pathological damage degree of pancreas increased evidently with the prolongation of HTG duration (Fig. 2A,B and Fig. S6).

Given the most serious pathological damage took place 28 days post P-407 injection, we chose this long-term HTG model to observe the dynamical changes of pancreas at 4, 8 and 12 h after standard dose Cae (50ug/kg) injection. Indeed, the pathological changes of P-407 + Cae group mice increased over time and were much higher than PBS + Cae group mice at each time point (Fig. S7). To our surprise, the pancreatic injuries of P-407 + Cae group mice at 8 h after Cae injection were even severer than those of PBS+Cae group mice at 12 h.

AP is a kind of disease in which pancreatic local inflammation progresses into severe systemic inflammatory responses, therefore, local and systemic inflammatory levels are commonly used to assess the severity of AP. We used ELISA to detect the serum levels of inflammatory cytokines as well as the levels in pancreatic tissue and it turned out to be consistent with the pathological results (Fig. 2C,D).

**Figure 3:**
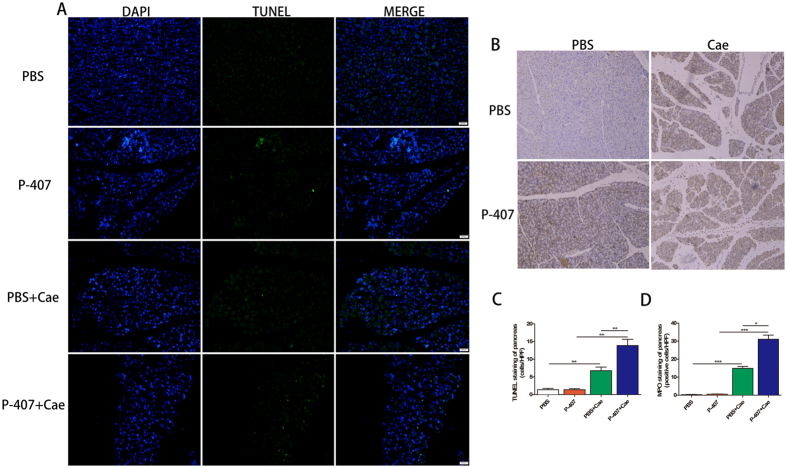
HTG was induced by long term 28 days P-407 (0.5 g/kg) injection, Mice was treated with standard dose Cae (50 ug/kg) to induced AP model. n = 6–8 each group. (**A**) Representative immunohistochemistry images for TUNEL staining in pancreatic tissue and (**B**) for MPO stained sections in pancreatic tissue. Immunohistochemistry stained sections of pancreas in magnification 100X. (**C**) The apoptotic cells counting of TUNEL staining. (**D**) The neutrophils counting of MPO staining. *P < 0.05, **P < 0.01, *** < 0.001.

Myeloperoxidase (MPO) is mainly expressed in neutrophils and could be used as a biomarker of activated neutrophils, Immunohistochemistry examination for MPO of pancreatic tissue indicated that P-407 + Cae group mice had more neutrophils infiltration compared with PBS + Cae group mice. (Figure 3B,D). Terminal deoxynucleotidyl transferase-mediated dUTP-biotin nick end labeling (TUNEL) staining study, which could detect cell apoptosis, revealed that pancreatic acinar cells in P-407 + Cae group were involved in more extensive apoptosis process (Fig. 3A,C).

In addition, to validate the above findings, we also adopted the Institute of Cancer Research (ICR) mice strains and the results match that in the C57BL/6 mice, but the inflammation degree of pancreas in ICR mice was severer than that in C57BL/6 mice, implying that ICR mice are more susceptible to AP than C57BL/6 mice (Fig. S4).

### HTG exacerbated the severity of acute lung injury in AP

**Figure 4:**
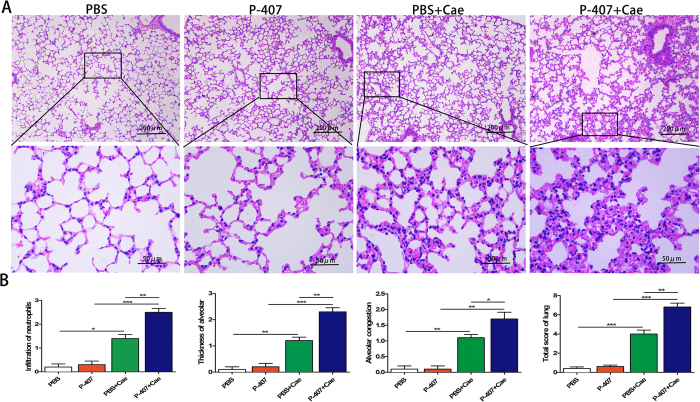
HTG exacerbated the severity of acute lung injury in AP in mice. HTG was induced by long term 28 days P-407 (0.5 g/kg) injection, Mice was treated with standard dose Cae (50 ug/kg) to induce AP model. n = 6–8 each group. (**A**) Representative pathological changes in lung tissues. HE stained sections of lung in magnification 100X and 400X. (**B**) Histological scores of pulmonary tissues. *P < 0.05, **P < 0.01, *** < 0.001.

Acute lung injury (ALI) is one of the most common complications of severe acute pancreatitis (SAP) and patients with SAP are usually required to have mechanical ventilation. Previous studies have indicated that the incidence of acute lung injury in patients with HTG-AP was significantly higher than that in other types of AP^18^,^20^. As expected, there were obvious pathological changes in lungs of P-407 + Cae group mice in comparison with the PBS + Cae group mice. A large number of inflammatory cell infiltration and capillary congestion in the alveolar septum were observed in lung of P-407 + Cae group mice and the total pathological scores of lung in P-407 + Cae group mice were significantly higher than that in PBS + Cae group mice (Fig. 4).

### HTG increased susceptibility to AP

**Figure 5:**
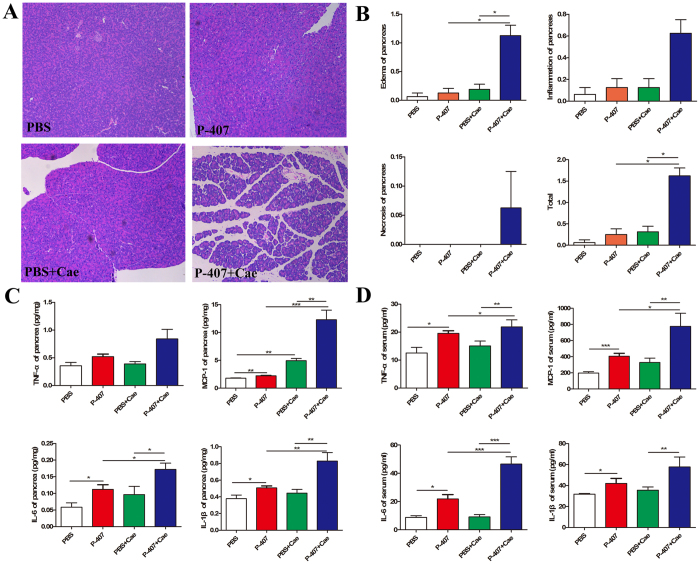
HTG increased susceptibility to AP in mice. HTG was induced by long term 28 days P-407 (0.5 g/kg) injection, Mice was treated with low dose Cae (5 ug/kg) to induce AP model. n = 6–8 each group. (**A**) Representative pathological changes in pancreas. HE stained sections of pancreas in magnification 100X. (**B**) Histological scores of pancreatic tissue. (**C**) Levels of TNF-a, IL-6, MCP-1, IL-1β in pancreatic tissues. (**D**) Serum levels of TNF-a, IL-6, MCP-1, IL-1β. *P < 0.05, **P < 0.01, *** < 0.001.

To explore the role of HTG in AP, we first treated normal mice with 10 intraperitoneal injections of Cae at five consecutive gradient doses (1 ug/kg, 2.5 ug/kg, 5 ug/kg, 10 ug/kg and 20 ug/kg b.w) at hourly intervals to induce AP. Histological examination results showed that pancreas of mice in 10 ug/kg group had obvious inflammatory changes (Fig. S8). While in 5 ug/kg group mice, apart from the slight elevation in monocyte chemotactic protein-1 (MCP-1) levels in pancreatic tissue (Fig. 5C), we failed to observe the typical pathological changes of AP and there was no significant difference in pathological scores between the 5 ug/kg group mice and the normal mice (Fig. 5A,B). Next we treated P-407 group mice with Cae at dose of 5 ug/kg b.w and obvious inflammatory cells infiltration and edema were observed in sections of pancreatic tissue (Fig. 5A,B and Fig. S9) along with the remarkable elevation of inflammatory cytokines levels both in the serum and pancreatic tissue (Fig. 5C,D), which demonstrated that HTG increases the susceptibility of mice to AP.

To strengthen the above findings, we treated P-407 group mice with Cae at doses of 10 ug/kg and 20 ug/kg b.w and PBS group mice with the Cae dose of 50 ug/kg b.w. There is no significant difference in total pathological changes between 20 ug/kg Cae induced P-407group mice and 50 ug/kg Cae induced PBS group mice (Fig. S10), suggesting HTG increases susceptibility to AP from the other side.

### HTG promoted the infiltration of macrophages and neutrophils in pancreas

**Figure 6:**
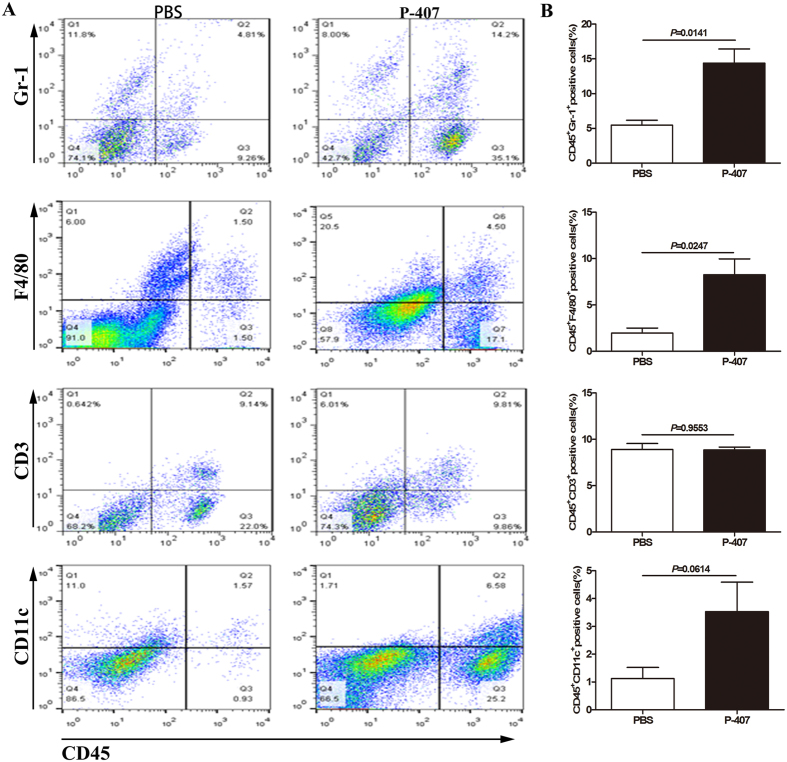
HTG promoted the infiltration of immune cells in pancreas in mice. HTG was induced by long term 28 days P-407 (0.5 g/kg) injection. n = 3–4 each group. (**A**) CD45^+^Gr-1^+^, CD45^+^F4/80^+^, CD45^+^CD3^+^, CD45^+^CD11c^+^ cells in the pancreatic tissues. (**B**) Numbers of the four types of cells of PBS group mice and P-407 group mice.

In order to explore the underlying mechanism of HTG increasing susceptibility to AP, we examined the alterations of immune cell states in pancreatic tissue. It had been observed that the percentages of CD45^+^F4/80^+^, CD45^+^Gr-1^+^ cells increased significantly after the long-term P-407 administration, which implying that HTG for a long time caused the infiltration of macrophages and neutrophils and generated local inflammatory microenvironment in pancreas, which this was in line with the elevated inflammatory cytokines levels in pancreatic tissue (Fig. 6).

## Discussion

Our study successfully established a novel mice model of HTG-AP with P-407 joint Cae. Through this model, we found that HTG can aggravate pancreas and lung injury under the condition of AP. Meanwhile, we for the first time put forward that HTG could increase susceptibility to AP from the aspect of animal experiments.

AP is common and fatal acute inflammation of pancreas, whose global incidence increased year by year. Although most cases only feature as mild inflammatory change, there are still about 20% cases developing into critical illness and the mortality rate exceeds 30%^21^. HTG is one of the most common etiologies of AP while the present studies concerning the mechanism of HTG-AP are in slow progress worldwide, which may be related to the lack of appropriate animal models. All the reported animal models of HTG-AP have defects to varying degrees.

In 1996, Friess *et al*.^22^ built a rat model of HTG-AP with TritonWR 1339 by tail vein injection, and plasma triglyceride level in this model could reach up to about 1000 mg/dl, but only lasting for 24 hours and it was difficult to keep the stable state of high blood triglycerides, what’s more, TritonWR 1339 was really expensive. LPL genetically deficient mink^23^,^24^ and high-fat diet hamster^25^ both existed HTG, but the absence of antibodies was not in favor of the immunology and mechanism research on AP^25^. Along with the development of gene modification technology in recent years, the problem that stable and effective HTG-AP model can’t be established simply relying on drugs or high fat diet, has been solved by genetically modified animals, such as LPL-deficient mice^9^,^10^,^11^ and ApoCIII-tg mice^12^,^13^. However, genetically modified animals still cannot be used widely because of high cost of breeding, difficult reproduction, mismatch with human plasma lipids and so on. Therefore, there is an urgent need to establish a novel stable, effective and simple HTG-AP model.

Our model simulated mice HTG with intraperitoneal injection of P-407, and then induced AP by intraperitoneal injection of Cae. Although P-407 is also a non-ionic surface active agent, like TritonWR 1339, but the physiological toxicity of P-407 is low and the plasma triglyceride level of mice can be maintained stably at 5000 mg/dl after the prolonged stimulation with P-407^14^. Compared with genetically modified animal models, this novel HTG model has the advantages of simple operation, low cost and plasma lipid matching with HTG-AP patients, consequently imitating the pathophysiological process of such patients well.

The clinical manifestations of HTG-AP have no significant differences with other types of AP, but generally it has worse prognosis, longer length of stay in hospital and higher morbidity rate of complications. Lindkvist *et al*.^8^ showed that HTG was an independent risk factor for persistent organ failure in patients with AP. Pathological examination in our study has demonstrated that severity of pancreatic injury, inflammatory cell infiltration and other pathological changes in the HTG group were significantly higher than those in the control group and positively correlated with the extents and durations of serum triglyceride levels.

More importantly, for the first time, in our study we proved that HTG could increase the susceptibility to AP in mice. Previous clinical studies stated that the serum pro-inflammatory cytokines were remarkably elevated in HTG patients^26^,^27^, consistently, Liu *et al*.^12^ who established HTG-AP model with ApoCIII-tg mice joint Cae, also declared that the monocyte migration and pancreatic injury, along with the expression of inflammatory cytokines, such as TNF-a, IL-6, MCP-1 of HTG group were significantly higher than those of the wild type mice.

Saja *et al*.^17^ also verified that long-term HTG could cause lipid deposition and macrophages infiltration in heart, liver and kidney. Our results were consistent with the conclusion of predecessors that we found that the pro-inflammatory cytokines TNF-a, IL-6, MCP-1,IL-1β both in blood and pancreatic tissues of the HTG mice were significantly higher than normal mice, which indicating that HTG mice possessed local and systemic inflammatory responses. At the same time, Flow Cytometry results manifested that HTG leaded to the increased infiltrations of macrophages and neutrophils in mice pancreatic tissues. Collectively, the pancreatic local inflammatory microenvironment and systemic inflammation predisposed the HTG mice to get AP and make AP severer. What’s more, we have sound reasons to believe that severer pancreatic injury, mononuclear cell migration and increasing expression of inflammation cytokines are common phenomenon of different HTG model, which implies that the pathophysiological changes of pancreas are caused mainly by HTG rather than the toxic effect of P-407.

In conclusion, P-407 joint Cae can build stable and controllable mice model of severe HTG-AP, at the same time, HTG can increase the susceptibility to AP and aggravate the injury of pancreas and lung under the condition of AP.

## Materials and Methods

### Animals and Diets

Male mice in C57BL/6 background weighing approximately 20–25 g were purchased from Model Animal Research Center of Nanjing University (Nanjing, China). All mice were housed in a SPF standard room under 12/12 h light-dark cycle at 24 °C, given water ad libitum, fed standard laboratory chow and were allowed to acclimatize for a minimum of 1 week. All methods were carried out in accordance with The Principles of L*aboratory Animal Care* (NIH publication no. 85Y23, revised 1996); All experimental protocols were approved by the experimental animal ethics committee of Jinling Hospital affiliated to medical School of Nanjing University (No. 20151008). Meanwhile, in order to verify whether the model is universal in different mice strains, male mice in ICR background weighing approximately 28–32 g were also used in this research.

### Experimental Design and Procedures

The HTG model was developed by administering P-407 (Pluronic F-127, Sigma-Aldrich Co., St. Louis, MO, USA) intraperitoneally to mice each other day at the dose level of 0.1, 0.25,0.5 g/kg body weight (b.w), the control group was administered PBS equivalently in the same amount. P-407 was mixed with phosphate buffered saline (PBS; pH = 7.4) and refrigerated at 4 °C overnight to dissolve completely. One single intraperitoneal injection of P-407 could establish the transient HTG model, short-term HTG model was established via 7 consecutive dosing days, and long-term HTG model is set up via 28 consecutive dosing days. C57BL/6 mice were randomly assigned to 4 groups: PBS, P-407, PBS + Cae and P-407 + Cae. AP was induced by 10 intraperitoneal injections of Cae (AnaSpec, Inc., Fremont, USA) b.w in PBS at hourly intervals, and the control group injected with PBS in the same way. Blood samples were obtained from the tail veins of isoflurane-anesthetized mice at different hours after the first Cae injection. Then animals were anaesthetized with an intraperitoneal administration of sodium pentobarbital (50 mg/Kg) and sacrificed, and pancreatic tissues, along with pulmonary tissues, were taken and fixed in 4% paraformaldehyde in PBS and embedded in paraffin.

### Measurement of Plasma Lipids and lipoprotein

Total cholesterol (TC) and triglyceride levels were determined with a commercially available kit (Beijing Zhongsheng Beikong Biochemistry Company, PR China) according to the manufacturer’s protocol. For determination of the lipids distributed in plasma lipoprotein, FPLC was performed with 200lL of pooled plasma from 10 mice per group, using a Superose 6 column (Amersham Bioscience) as described previously^28^. Forty fractions of 0.5 mL each were collected and enzymatically assayed for TC and triglyceride content.

### Plasma Biochemical Assay

The plasma of mice was centrifuged at 28,000 rpm for 30 minutes at 4 °C to remove chylomicrons. Amylase activity was measured by 5-ethylidene-G_7_PNP as a substrate with a commercial kit (Beijing Zhongsheng Beikong Biochemistry Company, PR China), and lipase activity was also measured with a commercial kit (Nanjing Jiancheng Biochemistry Company, PR China), as described in the manual from the manufacturer. Serum alanine-aminotransferase (ALT), total bilirubin (TBIL), creatinine (Cr) and urea nitrogen (BUN) were measured with dry chemistry method in General Surgery Biochemistry Laboratory of Jinling Hospital (AU680 automatic biochemical analysis system, Beckman Coulter Inc., USA). Serum ApoCIII levels (Cloud-Clone Crop., Wuhan, PR China) and free fatty acids(Wako Pure Chemical Industries, Ltd., Osaka, Japan) were determined according to the manual from the manufacturer.

### Histological Examination

The Paraffin sections of pancreas and lung tissue were stained with hematoxylin and eosin. Two investigators who were blind to the experimental treatment scored the degree of pancreatic injury by light microscopy, evaluating the severity of edema, inflammation and necrosis, as we described previously in Table 1^25^,^26^. We also scored the degree of pulmonary injury by evaluating the severity of neutrophil infiltration, thickness of alveolar and alveolar congestion, and the scoring standards were described previously as in Table 2^27^.

### Inflammatory cytokines measurement

Briefly, we homogenated pancreatic tissue in PBS and then carried out centrifugation (12000 rpm, 4 °C, 30 min) to get supernatant, the serum TNF-a, IL-6, MCP-1,IL-1β levels were measured with a commercial kit (Affymetrix ebioscience, Santiago, USA).

### Immunohistochemical Examination and TUNEL Staining

The slices from paraffin-embedded tissues were subjected to immunohistochemical staining for myeloperoxidase (MPO). The prepared slices were washed in PBS for 10 min and then boiled in 0.01 mmol citrate buffer (pH = 6) for 10 min for antigen retrieval. After incubation with hydrogen peroxide for 10 min, 5% bovine serum albumin (BSA) was applied as the blocking solution for 20 min at room temperature. Without washing, the sections were incubated with anti-Myeloperoxidase antibody (1:100) (ab9535, Abcam, Cambridge, UK) overnight at 4 °C. After being rinsed with PBS, the sections were incubated with goat anti-rabbit secondary antibody (1:500) (ab150079, Abcam, Cambridge, UK) and then visualised using a 3, 3-diaminobenzidine (DAB) kit (AR1022, Boster, Wuhan, China). Finally, images were recorded using a microscope at 100× magnification (IX73, Olympus, Tokyo, Japan). The TUNEL staining for apoptosis operation was performed with a commercial cell death detection kit purchased from Roche Diagnostics (Indianapolis, USA) according to the manufacturer’s protocol. The stained slices were observed by microscopy (IX73, Olympus, Tokyo, Japan) and images were recorded.

### Isolation of pancreatic immune cells of mice and Flow Cytometry

Pancreatic immune cells were isolated using collagenase IV digestion method described by J Xue. *et al*. for flow cytometry analysis^29^. All antibodies used for flow cytometry were purchased from BD Biosciences, unless indicated. For surface staining, cells were collected and stained with CD3, CD45, CD 11 C, F4/80, Gr-1 antibodies. The labeled cells were analyzed by flow cytometry using CellQuest (BD FACSCalibur) or FACS Diva (BD FACSAria software).

### Statistical Analysis

Statistical analysis was performed by SPSS 22.0 software. Results are presented as mean ± standard deviation (SD). The data of biochemistry measurements were analyzed with a one-way analysis of variance and the Student-Newman-Keuls test. In the histological evaluation, the results were analyzed by a Mann-Whitney rank sum test, and P < 0.05 was considered statistically significant.

## Additional Information

**How to cite this article**: Pan, Y. *et al*. Development of a novel model of hypertriglyceridemic acute pancreatitis in mice. *Sci. Rep.* **7**, 40799; doi: 10.1038/srep40799 (2017).

**Publisher's note:** Springer Nature remains neutral with regard to jurisdictional claims in published maps and institutional affiliations.

## Supplementary Material

Supplementary Information

## Figures and Tables

**Table 1:** Histopathological Scoring of Pancreatic injury.

Score	Edema	Acinar necrosis	Inflammation
0	Absent	Absent	0–5 leukocytes/HPF
1	Diffuse expansion of interlobar septae	1–4 necrotic cells/HPF	6–15 leukocytes/HPF
2	Diffuse expansion of interlobubar septae	5–10 necrotic cells/HPF	16–25 leukocytes/HPF
3	Diffuse expansion of interacinar septae	11–16 necrotic cells/HPF (foci of confluent necrosis)	26–35 leukocytes/HPF
4	Diffuse expansion of intercellular spaces	>16 necrotic cells/HPF (extensive confluent necrosis)	>35 leukocytes/HPF or confluent microabscesses

Acinar cell necrosis and inflammatory infiltration were counted as the average number per 10 fields at magnification 400×.

**Table 2:** Histopathological Scoring of Lung injury.

Score	Thickness of alveolar	Infiltration of neutrophils	Alveolar congestion
0	Absent	Absent	Absent
1	Discrete	Discrete	Small foci
2	Moderate	Moderate	Large foci
3	Severe	Severe	Diffuse

## References

[b1] Tenner S., Baillie J., DeWitt J., Vege S. S. & American College of, G. American College of Gastroenterology guideline: management of acute pancreatitis. The American journal of gastroenterology 108, 1400–1415; 1416, doi: 10.1038/ajg.2013.218 (2013). 23896955

[b2] Karsidag T., Tuzun S. & Makine C. Domino effect from hypertriglyceridemia to sinistral portal hypertension. Chirurgia (Bucharest, Romania: 1990) 104, 219–222 (2009). 19499667

[b3] Gullo L. . Acute pancreatitis in five European countries: etiology and mortality. Pancreas 24, 223–227 (2002). 11893928 10.1097/00006676-200204000-00003

[b4] Scherer J., Singh V. P., Pitchumoni C. S. & Yadav D. Issues in hypertriglyceridemic pancreatitis: an update. Journal of clinical gastroenterology 48, 195–203, doi: 10.1097/01.mcg.0000436438.60145.5a (2014). 24172179 PMC3939000

[b5] Valdivielso P., Ramirez-Bueno A. & Ewald N. Current knowledge of hypertriglyceridemic pancreatitis. European journal of internal medicine 25, 689–694, doi: 10.1016/j.ejim.2014.08.008 (2014). 25269432

[b6] Huang Y. X. . Incidence and clinical features of hyperlipidemic acute pancreatitis from Guangdong, China: a retrospective multicenter study. Pancreas 43, 548–552, doi: 10.1097/MPA.0000000000000069 (2014). 24717803

[b7] Christian J. B., Bourgeois N., Snipes R. & Lowe K. A. Prevalence of severe (500 to 2,000 mg/dl) hypertriglyceridemia in United States adults. The American journal of cardiology 107, 891–897, doi: 10.1016/j.amjcard.2010.11.008 (2011). 21247544

[b8] Lindkvist B., Appelros S., Regner S. & Manjer J. A prospective cohort study on risk of acute pancreatitis related to serum triglycerides, cholesterol and fasting glucose. Pancreatology : official journal of the International Association of Pancreatology (IAP) … [et al.] 12, 317–324, doi: 10.1016/j.pan.2012.05.002 (2012). 22898632

[b9] Tang M. . A serum metabolomic investigation on lipoprotein lipase-deficient mice with hyperlipidemic pancreatitis using gas chromatography/mass spectrometry. Biomedical reports 1, 469–473, doi: 10.3892/br.2013.78 (2013). 24648970 PMC3916981

[b10] Wang Y. . Enhanced susceptibility to pancreatitis in severe hypertriglyceridaemic lipoprotein lipase-deficient mice and agonist-like function of pancreatic lipase in pancreatic cells. Gut 58, 422–430, doi: 10.1136/gut.2007.146258 (2009). 18936103

[b11] Yang F. . The role of free fatty acids, pancreatic lipase and Ca+ signalling in injury of isolated acinar cells and pancreatitis model in lipoprotein lipase-deficient mice. Acta physiologica 195, 13–28, doi: 10.1111/j.1748-1716.2008.01933.x (2009). 18983441

[b12] Liu J. . FTY720 Attenuates Acute Pancreatitis in Hypertriglyceridemic Apolipoprotein CIII Transgenic Mice. Shock 44, 280–286, doi: 10.1097/SHK.0000000000000400 (2015). 25944794

[b13] Ehx G. . Liver proteomic response to hypertriglyceridemia in human-apolipoprotein C-III transgenic mice at cellular and mitochondrial compartment levels. Lipids in health and disease 13, 116, doi: 10.1186/1476-511X-13-116 (2014). 25047818 PMC4112841

[b14] Sharyo S., Kumagai K., Yokota-Ikeda N., Ito K. & Ikeda M. Amelioration of renal ischemia-reperfusion injury by inhibition of IL-6 production in the poloxamer 407-induced mouse model of hyperlipidemia. Journal of pharmacological sciences 110, 47–54 (2009). 19403996 10.1254/jphs.08283fp

[b15] Wasan K. M. . Poloxamer 407-mediated alterations in the activities of enzymes regulating lipid metabolism in rats. Journal of pharmacy & pharmaceutical sciences: a publication of the Canadian Society for Pharmaceutical Sciences, Societe canadienne des sciences pharmaceutiques 6, 189–197 (2003). 12935429

[b16] Johnston T. P. The P-407-induced murine model of dose-controlled hyperlipidemia and atherosclerosis: a review of findings to date. Journal of cardiovascular pharmacology 43, 595–606 (2004). 15085072 10.1097/00005344-200404000-00016

[b17] Saja M. F. . Triglyceride-Rich Lipoproteins Modulate the Distribution and Extravasation of Ly6C/Gr1(low) Monocytes. Cell reports 12, 1802–1815, doi: 10.1016/j.celrep.2015.08.020 (2015). 26344769 PMC4590546

[b18] Nawaz H. . Elevated serum triglycerides are independently associated with persistent organ failure in acute pancreatitis. The American journal of gastroenterology 110, 1497–1503, doi: 10.1038/ajg.2015.261 (2015). 26323188

[b19] Zhang X. L., Li F., Zhen Y. M., Li A. & Fang Y. Clinical Study of 224 Patients with Hypertriglyceridemia Pancreatitis. Chinese medical journal 128, 2045–2049, doi: 10.4103/0366-6999.161361 (2015). 26228216 PMC4717952

[b20] Lechleitner M. . [Hypertriglyceridemia and acute pancreatitis]. Acta medica Austriaca 21, 125–128 (1994). 7709709

[b21] Famularo G., Minisola G. & De Simone C. Acute pancreatitis. The New England journal of medicine 355, 961; author reply 961, doi: 10.1056/NEJMc061618 (2006). 16943414

[b22] Hofbauer B. . Hyperlipaemia intensifies the course of acute oedematous and acute necrotising pancreatitis in the rat. Gut 38, 753–758 (1996). 8707124 10.1136/gut.38.5.753 PMC1383160

[b23] Lindberg A. . A mutation in the lipoprotein lipase gene associated with hyperlipoproteinemia type I in mink: studies on lipid and lipase levels in heterozygotes. International journal of molecular medicine 1, 529–538 (1998). 9852258 10.3892/ijmm.1.3.529

[b24] Nordstoga K. . Pancreatitis associated with hyperlipoproteinaemia type I in mink (Mustela vison): earliest detectable changes occur in mitochondria of exocrine cells. Journal of comparative pathology 134, 320–328, doi: 10.1016/j.jcpa.2006.01.003 (2006). 16709420

[b25] Hu G. . Development of a novel model of hypertriglyceridemic acute pancreatitis in hamsters: protective effects of probucol. Pancreas 41, 845–848, doi: 10.1097/MPA.0b013e318247d784 (2012). 22781908

[b26] Bosques-Padilla F. J. . Hypertriglyceridemia-induced pancreatitis and risk of persistent systemic inflammatory response syndrome. The American journal of the medical sciences 349, 206–211, doi: 10.1097/MAJ.0000000000000392 (2015). 25545390

[b27] Mirhafez S. R. . Association between serum cytokine concentrations and the presence of hypertriglyceridemia. Clinical biochemistry 49, 750–755, doi: 10.1016/j.clinbiochem.2016.03.009 (2016). 27048855

[b28] Wei J. . Characterization of a hypertriglyceridemic transgenic miniature pig model expressing human apolipoprotein CIII. The FEBS journal 279, 91–99, doi: 10.1111/j.1742-4658.2011.08401.x (2012). 22023023

[b29] Xue J. . Alternatively activated macrophages promote pancreatic fibrosis in chronic pancreatitis. Nature communications 6, 7158, doi: 10.1038/ncomms8158 (2015). PMC4632846 25981357

