# Supplementary material for: Development of a novel model of hypertriglyceridemic acute pancreatitis in mice
Source: Sci Rep. 2017 Jan 12;7:40799. doi: 10.1038/srep40799 (PMC5228057; doi:10.1038/srep40799)
Supplement: Supplementary Information [file srep40799-s1.pdf]

Development of a novel model of hypertriglyceridemic acute pancreatitis in mice

Yiyuan Pan[1#], Yong Li[1#], Lin Gao[1#], Zhihui Tong[1], Bo Ye[1], Shufeng Liu[2], Baiqiang Li[1], Yizhe Chen[1], Qi Yang[1], Lei Meng[1], Yuhui Wang[3], George Liu[3], Guotao Lu[1,4,*], and Weiqin Li[1,*], Jieshou Li[1]

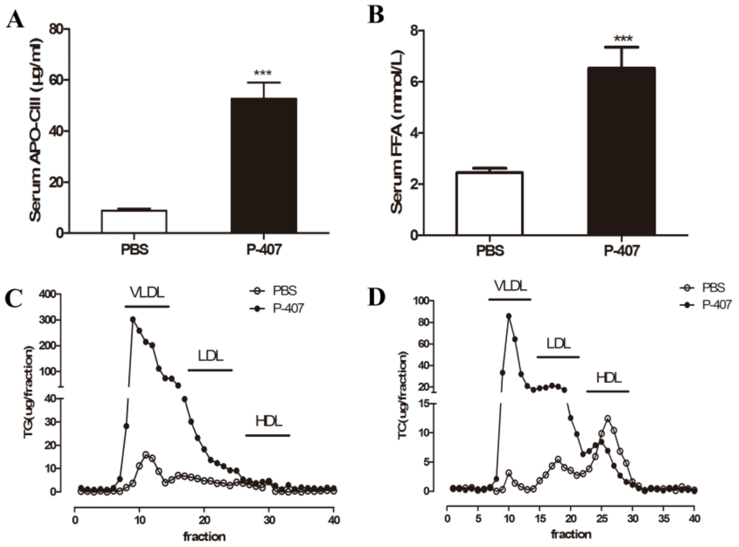

(Supplementary Figure S1) **P-407 altered lipid metabolism in mice.** The P-407 group mice were administrated with long-term 28 days P-407 (0.5g/kg) injections. n=8-10 each group. (A) The serum ApoCIII levels and (B) The serum free fat acids (FFAs) levels of the PBS group mice and P-407 group mice. (C, D) The serum lipoprotein distributions detected by FPLC. *P<0.05, **P<0.01, ***<0.001.

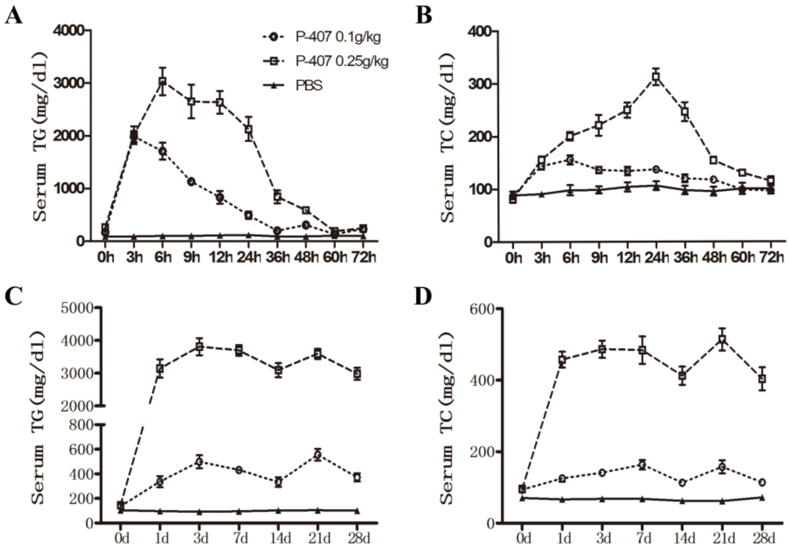

(Supplementary Figure S2) **The levels of HTG were positively correlated with the doses of P-407**. The changes of serum triglyceride and cholesterol levels of mice (A, B) after one single intraperitoneal injection of P-407 (0.1g/kg, 0.25g/kg) and PBS or (C, D)after 28 days injections of P-407 (0.1g/kg, 0.25g/kg) and PBS. n=8 each group.

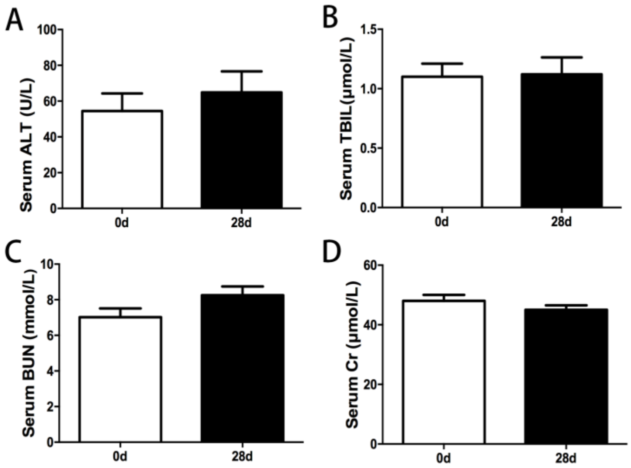

(Supplementary Figure S3) Serum alanine transaminase level, total bilirubin level, creatinine level and blood urea nitrogen level of P-407(0.5g/kg) induced mice at day0 and day28. n=10-12each group.

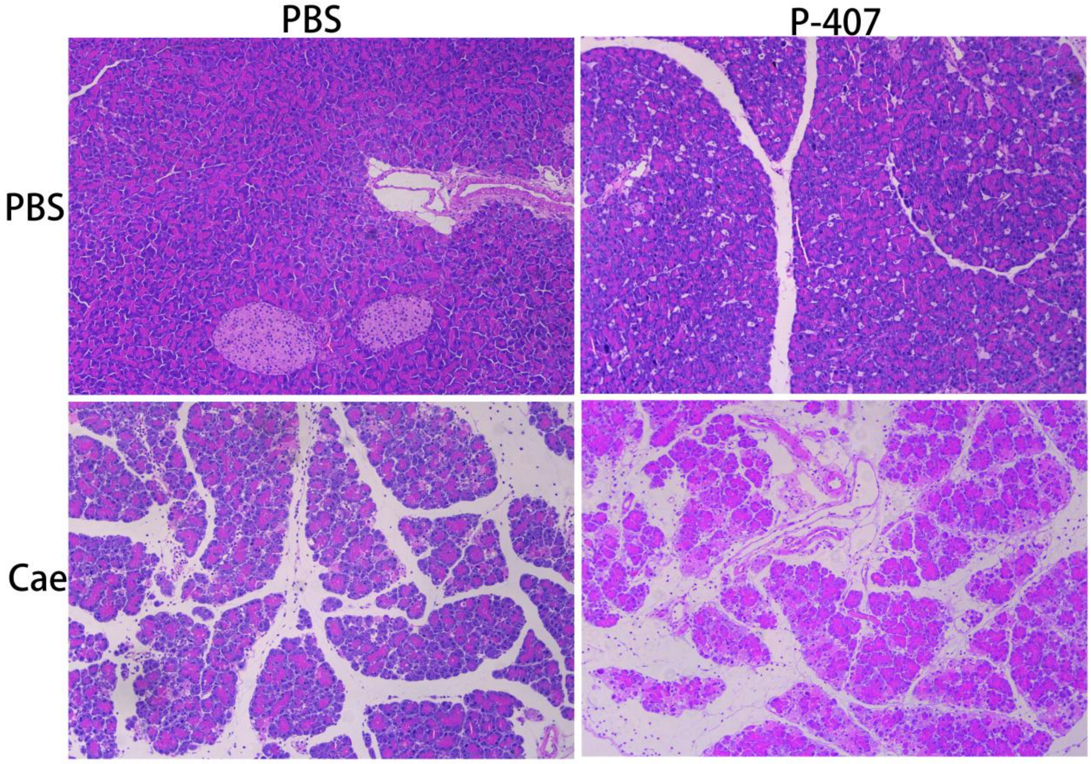

(Supplementary Figure S4) HTG was induced by long term 28 days P-407 (0.5g/kg) injection, ICR mice was treated with standard dose Cae (50ug/kg) to induce AP model. n=6-8 each group. Representative pathological changes in pancreas of four groups: PBS group, P-407 group, PBS+ Cae group and P-407+ Cae group. HE stained sections of pancreas in magnification 100X.

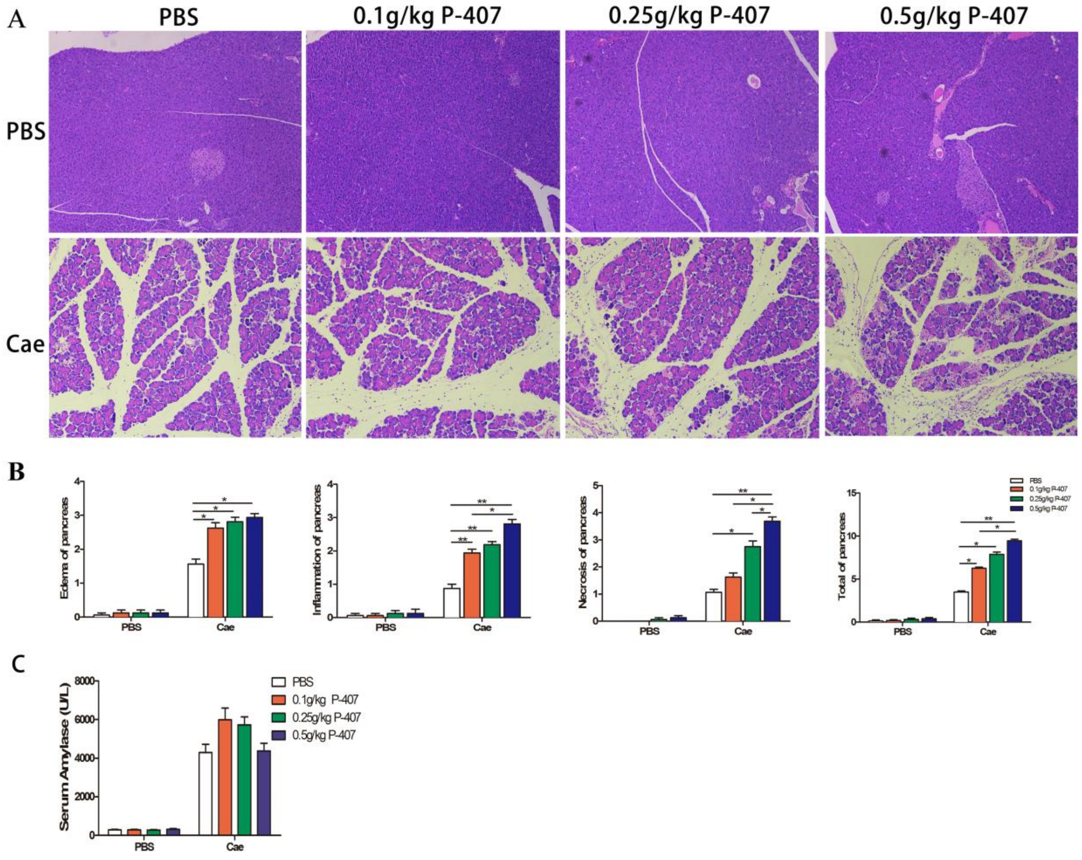

(Supplementary Figure S5) **The severities of AP were correlated with the extents of HTG.** HTG was induced by long term 28 days of different doses P-407 (0.1, 0.25, 0.5g/kg) injections, Mice was treated with standard dose Cae (50ug/kg) to induce AP model. n=8 each group. (A) Representative pathological changes of pancreatic tissue. HE stained sections of pancreas in magnification 100X. (C)Serum levels of amylase. *P<0.05, **P<0.01, ***<0.001.

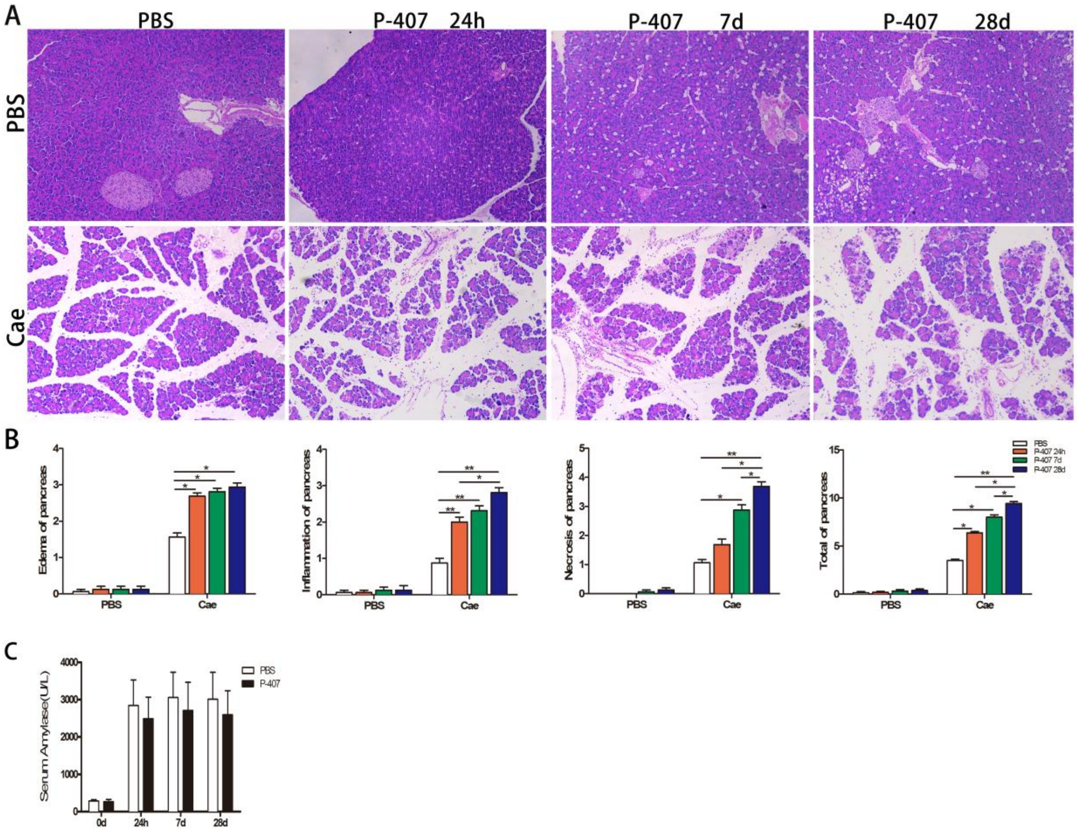

(Supplementary Figure S6) **The severities of AP were correlated with the durations of HTG**.HTG was induced by different durations of P-407 (0.5g/kg) injections, Mice was treated with standard dose Cae (50ug/kg) to induce AP model. n=8-12 each group. (A) Representative pathological changes of pancreatic tissue. HE stained sections of pancreas in magnification 100X. (C)Serum levels of amylase. *P<0.05, **P<0.01, ***<0.001.

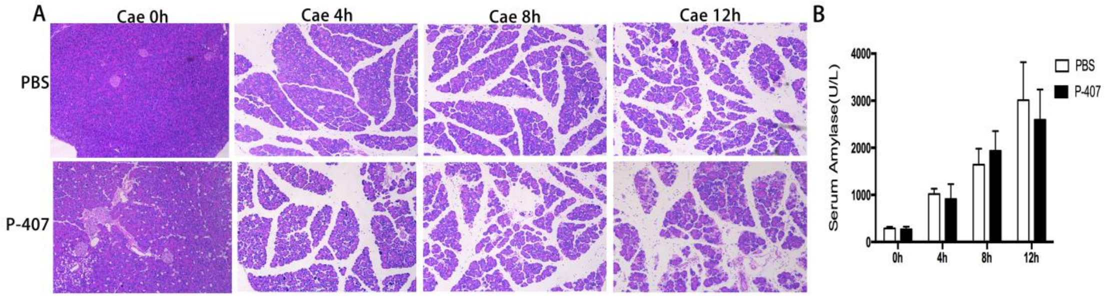

(Supplementary Figure S7) HTG was induced by long term 28 days P-407 (0.5g/kg) injections, Mice was treated with standard dose Cae (50ug/kg) to induce AP model. n=8 each group. (A) Representative pathological changes in pancreas at 4, 8 and 12h. HE stained sections of pancreas in magnification 100X. (B) The serum amylase levels.

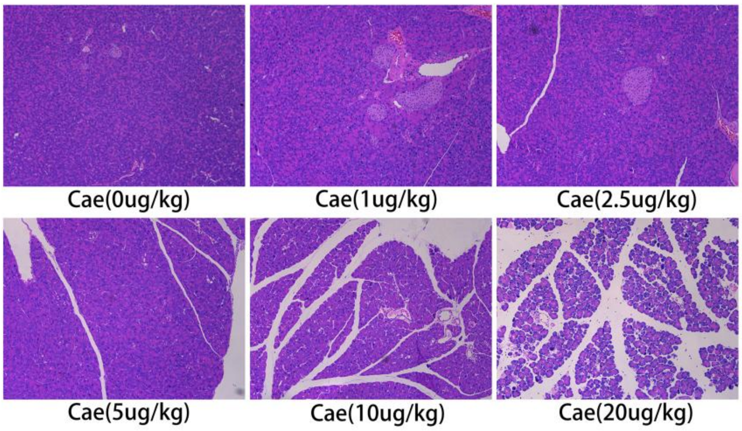

(Supplementary Figure S8) Representative pathological changes in pancreas of normal mice with 10 intraperitoneal injections of Cae at five consecutive gradient doses (1ug/kg, 2.5ug/kg, 5ug/kg, 10ug/kg and 20ug/kg b.w). n=6-8 each group.

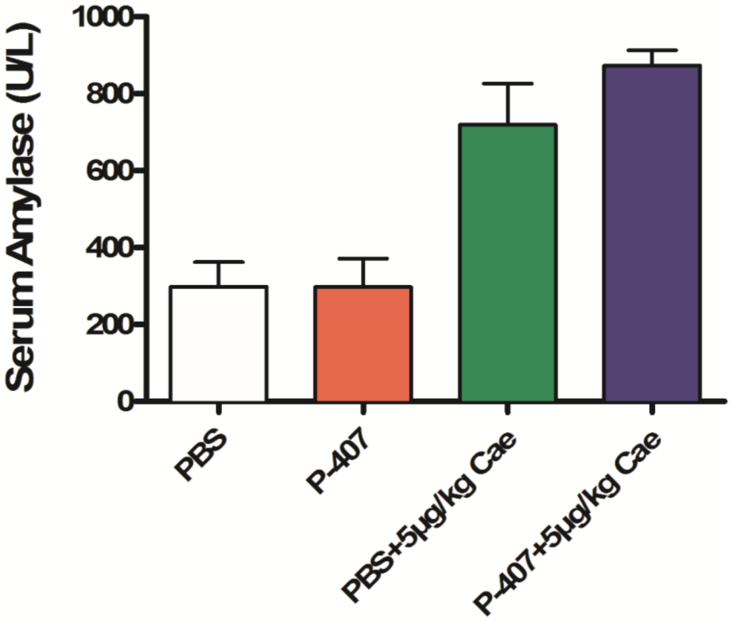

(Supplementary Figure S9) HTG was induced by long term 28 days P-407 (0.5g/kg) injections, Mice was treated with standard dose Cae (5ug/kg) to induce AP model. The serum amylase levels of the four groups: PBS group, P-407 group, PBS+Cae group and P-407+Cae group. n=6-8 each group.

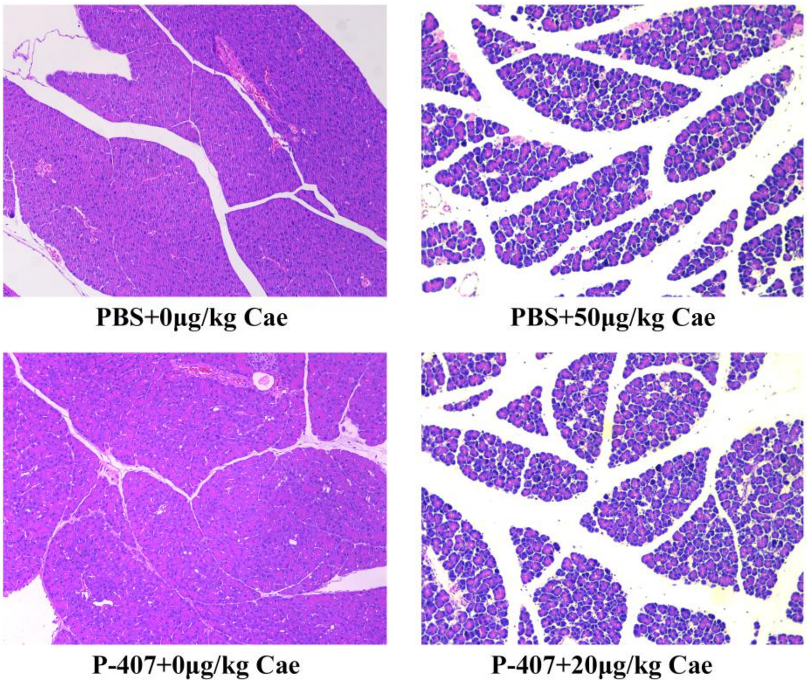

(Supplementary Figure S10) Representative pathological changes in pancreas of P-407 group mice treated with Cae at doses of 20ug/kg b.w and PBS group mice treated with the Cae dose of 50ug/kg b.w.
